# Lipid Profile Characterization of Human Micro-Fragmented Adipose Tissue via Untargeted Lipidomics

**DOI:** 10.3390/biom15070964

**Published:** 2025-07-04

**Authors:** Camillo Morano, Michele Dei Cas, Giulio Alessandri, Valentina Coccè, Francesca Paino, Monica Bignotto, Luisa Doneda, Carlo Tremolada, Augusto Pessina, Rita Paroni

**Affiliations:** 1 Clinical Biochemistry and Mass Spectrometry Laboratory, Department of Health Sciences, University of Milan, 20122 Milan, Italy; michele.deicas@unimi.it (M.D.C.); rita.paroni@unimi.it (R.P.); 2 CRC StaMeTec, Department of Biomedical, Surgical and Dental Sciences, University of Milan, 20133 Milan, Italy; giulio.alessandri@image.it (G.A.); valentina.cocce@unimi.it (V.C.); luisa.doneda@unimi.it (L.D.); augusto.pessina@unimi.it (A.P.); 3 Image Regenerative Clinic, 20122 Milan, Italy; carlo.tremolada@image.it; 4 Liver and Gastroenterology Unit, Department of Health Sciences, University of Milan, 20122 Milan, Italy; monica.bignotto@unimi.it

**Keywords:** micro-fragmented adipose tissue, fat tissue, lipidomics, drug delivery, mass spectrometry

## Abstract

Mesenchymal stem cells (MSCs) exhibit low immunogenicity, multipotency, and are abundantly present in adipose tissue, making this tissue an easily accessible resource for regenerative medicine. Different commercial procedures have been developed to micro-fragment the adipose tissue aspirate from patients before its reinjection. We explored a commercial device which mechanically micro-fragments human lipoaspirate (LA) resulting in a homogeneous micro-fragmentation of fat tissue (MFAT). This device has been successfully employed in several clinical applications involving autologous adipose tissue transplantation. Here, we compare the untargeted/targeted lipidomic profile of LA and MFAT looking for differences in terms of qualitative modifications occurring during the handling of the original LA material. In MFAT, different lipid subclasses such as diacylglycerols, triacylglycerols, phospholipids, and sphingolipids are more represented than in LA. In addition, via targeted fatty acids analysis, we found a lower abundance of monounsaturated fatty acids in MFAT. The biological implications of these findings must be better investigated to contribute to a better understanding of the clinical efficacy of MFAT and for its potential use as a scaffold for drug delivery applications.

## 1. Introduction

Since the beginning of the new millennium, interest in regenerative medicine has grown steadily. Many clinical fields are taking advantage of the most recent discoveries, such as esthetic medicine, orthopedics, cardiovascular surgery, cancer treatment, and dentistry [1,2].

Particularly, mesenchymal stem cells (MSCs) have gained the attention of the scientific community because of their potential differentiation capacity in numerous human adult tissues (i.e., bone, cartilage, adipose, muscle, and nerve) and are thus suitable for treating various conditions [3,4,5,6]. Furthermore, MSCs show low immunogenicity coupled with multipotency, allowing them to be harvested and reimplanted in the same subject as autologous transplants [7]. While harvesting MSCs from bone marrow is the gold standard for their high cell yield and proliferative capacity, this procedure is invasive and painful, and can be entirely avoided when using adipose-derived MSCs, with adipose tissue often available as a by-product of surgical procedures [8,9]. MSCs can be isolated from lipoaspirate through different processing methods. One common method involves the enzymatic dissociation of the tissue into single-cell suspensions, followed by centrifugation to remove adipocytes and isolate the stromal vascular fraction (SVF). While effective, enzymatic processing is time-consuming, costly, and subject to stricter regulatory constraints regarding cell manipulation (i.e., GMP procedure). As an alternative, mechanical processing techniques have been developed to produce micro-fragmented adipose tissue (MFAT) without the use of enzymes. MFAT preserves the native microenvironment and retains the SVF, which may enhance graft retention and therapeutic outcomes [10,11,12,13].

MFAT has gained popularity for the intra-articular treatment of knee osteoarthritis; however, few comparative studies exist between MFAT and other biologics. Additionally, MFAT products from different systems may vary in cellular composition, potentially influencing cytokine profiles and clinical efficacy—an aspect that remains poorly understood.

The current literature is further limited by heterogeneity in study quality and a lack of standardized methodologies. A recent systematic review and meta-analysis comparing MFAT to orthobiologics, hyaluronic acid, and corticosteroids for knee osteoarthritis found no statistically significant differences in pain relief or clinical outcomes [14]. This underscores the need for more rigorous studies and product standardization. One widely used mechanical processing method is the Lipogems^®^ system, which converts lipoaspirate into MFAT through mild mechanical forces. This process reduces the adipose tissue cluster size while removing pro-inflammatory oil and blood residues [10,15,16,17,18,19,20]. MFAT produced by this system retains structural similarity to native adipose tissue and shows enrichment in anti-inflammatory mediators, along with reduced contamination [21]. Such MFAT also shows potential as a drug delivery system, especially for agents with narrow therapeutic windows or high toxicity, such as chemotherapeutics [22,23]. Standardizing MFAT characterization techniques is essential to enable meaningful cross-study comparisons and to optimize the clinical applications of adipose-derived therapies. Several studies have demonstrated that the cellular content of MFAT depends on the processing method used. Thus, assessing graft cellularity with reproducible and comparable techniques is crucial for interpreting inter-study variability [24].

Given MFAT’s promising regenerative and therapeutic properties, there is a compelling need to thoroughly characterize its lipid composition, particularly to understand the biochemical changes induced by the micro-fragmentation process. This knowledge could unlock new applications, including its use as a scaffold for the sustained and safe release of active and potentially toxic drugs, such as those used in cancer chemotherapy [22]. Furthermore, investigating the lipidomic differences between MFAT and unprocessed lipoaspirate (LA) may inform future research and clinical innovation.

To our knowledge, this is the first study to analyze and compare the lipid profiles of human adipose tissue obtained via liposuction (LA) and its mechanically processed MFAT derivative. Our findings reveal quantitative differences in lipid content, particularly an increase in lipid subclasses such as diacylglycerols, triacylglycerols, and sphingolipids in MFAT.

## 2. Materials and Methods

### 2.1. Sample Collection

Samples of lipoaspirate (LA) were obtained by the liposuction of subcutaneous tissue using disposable cannulas provided with the Lipogems^®^ kit. Tissue samples were collected from five (n = 5) bariatric surgery operations—women with a mean age of 63 years—after the patient gave informed consent, in accordance with the Declaration of Helsinki. The approval for their use was obtained from the Institutional Ethical Committee of Milan University (n.59/15, C.E.UNIMI, 09.11.15).

### 2.2. LA and MFAT Preparation

**Figure 1:**
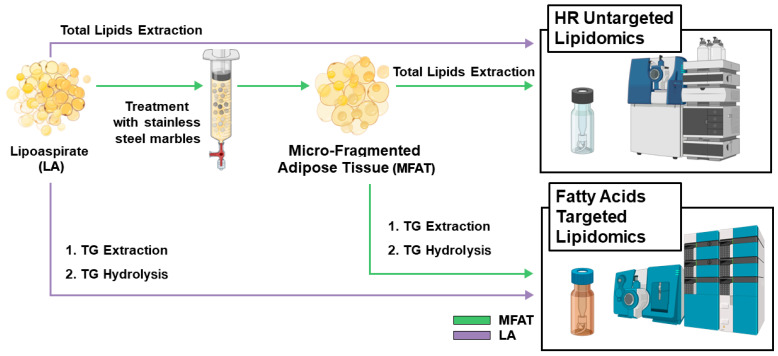
Micro-fragmented adipose tissue (MFAT) and lipoaspirate (LA) preparation and the analytical approaches used to investigate their lipidomic profile and the fatty acids profile derived from triglycerides hydrolysis.

MFAT specimens were prepared from LA as previously described [15]. Briefly, around 50/100 mL of LA was used for MFAT preparation using a standard 225 mL Lipogems^®^ device (provided by Lipogems^®^ International, Milan, Italy). The LA collected by the syringe was pushed into the Lipogems^®^ device through a filter for the first cluster reduction. Afterwards, the five stainless steel marbles inside the device were shaken to disaggregate fat materials, thus producing cell clusters and micro-fragmented fat tissue that migrated to the top of device, while blood-contaminating cells and undesired fat residues were removed by a gravity counterflow of saline solution. When the solution inside the device appeared yellow and clear, the device was turned upside down and a second micro-fragmentation of the fat clusters was performed through an additional filter with a grid of smaller pores. At the end of this procedure, MFAT product was aspirated with a syringe connected with the device and was ready for analysis. Figure 1 displays the complete experimental process adopted in this research from the preparation of MFAT to the analytical approaches employed to characterize its lipidomic profile.

### 2.3. Lipid Extraction from LA and MFAT

Lipid extraction was carried out according to the protocols outlined by Morano et al. in 2022 [25]. A small, weighted quantity of either LA or MFAT (10–20 mg) was combined with 100 µL of water, followed by the addition of 850 µL of a methanol/chloroform mixture (2:1, *v*/*v*). The mixture was then subjected to sonication and extraction in a thermomixer for 1 h (1000 RPM, 4 °C). Subsequently, the organic phase was separated through centrifugation (25 min at 15,000× *g*) and evaporated using a SpeedVac vacuum concentrator (Thermo Fisher, Waltham, MA, USA). The resulting residues were dissolved in 250 µL of isopropanol/acetonitrile containing 0.5 mg/mL butylated hydroxytoluene (BHT, 2:1 *v*/*v*). After centrifugation for 10 min at 15,000× *g*, the samples were transferred into glass vials.

### 2.4. LC-MS/MS Untargeted Method

LC-MS/MS analysis was conducted using a Shimadzu UPLC system coupled with a Triple TOF 6600 Sciex instrument (Concord, ON, Canada). Duplicate analyses of all samples were performed using both positive and negative electrospray ionization modes. The source parameters were set as follows: CUR, 35; GS1, 55; GS2, 65; capillary voltage, 5.5 kV (ESI+) or 4.5 kV (ESI-); and source temperature (TEM), 350 °C. Spectra were acquired simultaneously via full-mass scan at m/z 200–1500 with a TOF MS accumulation time of 100 ms and the top 20 data-dependent acquisitions at m/z 50–1500 with a TOF MS/MS accumulation time of 40 ms. The declustering potential was maintained at 50 eV, and the collision energy was set at 35 ± 15 eV. Chromatographic separation was achieved using a reversed-phase Acquity CSH C18 column (1.7 μm, 2.1 × 100 mm, Waters, Franklin, MA, USA) with a gradient elution system consisting of (A) water/acetonitrile (60:40) and (B) 2-propanol/acetonitrile (90:10), both containing 10 mM ammonium acetate and 0.1% formic acid. The flow rate was set at 0.4 mL/min, and the column temperature was maintained at 55 °C. The elution gradient (%B) was programmed as follows: 0–2.0 min (40%), 2.0–2.5 min (40–50%), 2.5–12.5 min (50–55%), 12.5–13.0 min (55–70%), 13.0–19.0 min (70–99%), 19.0–24.0 min (99%), and 24.0–24.2 min (99–40%), with %B held constant until 30 min. Five microliters of the clear lipid extract from LA and MFAT were directly injected into the LC-MS/MS system. [25]. All samples were analyzed in one analytical batch only and spaced out by samples pool, in order to minimize the instrumental variability as much as possible.

### 2.5. Lipidomic Data Processing

The spectra deconvolution, peak alignment, and sample normalization were attained using MS-DIAL (ver. 4.0, [26]). MS and MS/MS tolerance for peak profiles were set to 0.01 and 0.05 Da, respectively. Identification was achieved matching spectra with LipidBlast database. The analytical drift, which generally occurs during batch analysis, was resolved by LOWESS normalization injecting the QC pool sample every three runs. Analytes with a CV% superior to 30% in the QC pool sample and those with an intensity comparable or slightly higher than blanks were excluded. In addition to the automatic identification carried out by MS-DIAL, we performed a supplementary filtering by MS/MS similarity calculation via reverse dot/product (>50%). Analytes lacking MS/MS fragmentation and without accurate MS/MS matching were also excluded. The polarity and adducts that yielded the better results in terms of intensity and number of detected species were used in each lipid class. After that, each analyte intensity was then normalized according to the weighed amount of the initial fat sample.

**Figure 2:**
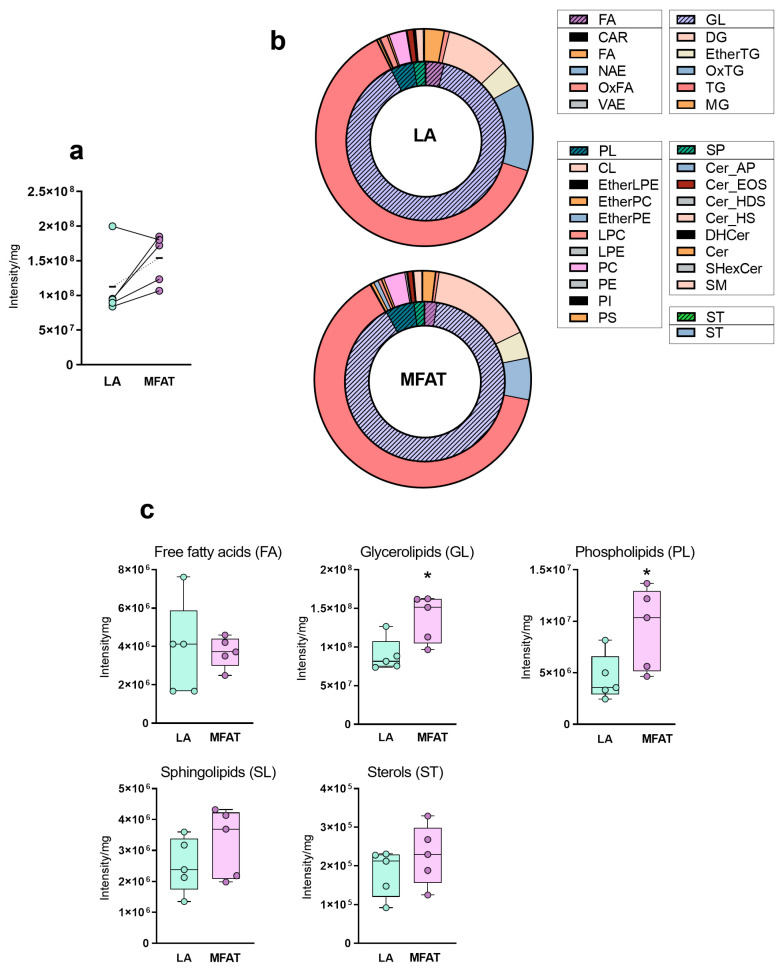
(**a**) Total lipid content in the adipose tissue lipoaspirate (LA, green) and after micro-fragmentation by the commercial device Lipogems (MFAT, purple) from five independent donors. Graphs represent the summed amounts of all recognized lipids, after normalization for mg of fat. Each point from the same subject is connected with a line. The dotted line connects the mean values of each group. The mean increase in lipid content of MFAT is about 37%. (**b**) Comparison of classes (inner donut) and subclasses (outer donut) distribution among LA and MFAT, expressed as % fraction of total lipid content. (**c**) Comparison of lipid classes content between LA and MFAT. Graphs represent the summed amounts (expressed as mass intensities), after normalization for mg of fat, of the individual lipids per class (Intensity, mean ± SD). Paired *t*-tests were used in each lipid class to establish statistical differences (* *p* < 0.05). FA: fatty acid derivatives; GL: glycerolipids; PL: phospholipids; SL: sphingolipids; ST: sterols.

Figure 2b (Section 3) shows an overview of the entire lipid distribution in both LA and MFAT: normalized lipid species intensities were grouped according to their classes (inner circles) and subclasses (outer circles).

### 2.6. Fatty Acids Profile After Triacylglycerols Hydrolysis of Lipoaspirate and MFAT

Triacylglycerols extraction was completed on a small portion of fat tissue (10–20 mg), which was added to 300 µL of water and extracted with 900 µL of ethyl acetate/diethyl ether/heptane mixture (1:1:1; *v*/*v*/*v*). Then it was sonicated and extracted in thermomixer for 1 h (1000 RPM, 4 °C). The organic phase was separated by centrifugation (15,000× *g*, 5 min) and evaporated under a stream of nitrogen. The residues were dissolved in 1000 µL of methanol and fatty acids from triacylglycerols were hydrolyzed by adding KOH in methanol (75 µL KOH 1M, 2 h 38°). The extracts were neutralized by adding pure acetic acid (4 µL). A portion of the extracts were exsiccated (100 µL) and reconstituted in isopropanol (100 µL). The fatty acids derived from triacylglycerol hydrolysis were then derivatized for 1 h at 38 °C with 3-NPH (50 µL 3-NPH 50 mM, 50 µL EDC 50 mM, and 50 µL 7% pyridine all the solutions were prepared in 70% MeOH). The samples were diluted with 0.5% FA in AcN/H_2_O (250 uL, 1:1, *v*/*v*) and injected in LC-MS/MS [27].

### 2.7. Statistical Analysis

For differences between the groups in the lipid metabolism, shown as the sum of the concentrations of the species within the class, data were compared by non-parametric *t*-test (Mann–Whitney) with GraphPad Prism 7.0 (GraphPad Software, Inc., La Jolla, CA, USA). For further statistical analysis, data tables with the lipids identified in the samples were formatted as comma-separated values (.csv) files and were uploaded to MetaboAnalyst 4.0 server. Data were checked for integrity, filtered by interquartile range, log transformed (generalized log transformation), and auto-scaled. Comparison between the two groups was performed by both univariate and multivariate unsupervised methods.

## 3. Results

### 3.1. Main Lipid Class Composition of Total Lipid Content

As previously mentioned, the aim of this work was to assess the potential differences in the qualitative and semi-quantitative lipidome composition that may arise in the MFAT material with respect to the original and less processed LA.

The first step was to evaluate the lipidome content via an untargeted approach, which enabled the assessment of the broader number of lipid species and revealed specific changes in the total lipid content of particular subclasses in five adipose tissue samples collected from five independent donors (see Section 2.1).

From the untargeted lipidome analysis, 3733 signals were attained, which, after data cleansing (see Material and Methods), were ascribed to 647 lipid species, belonging to 40 different subclasses common to all samples. These subclasses were then grouped in 5 classes, namely fatty acids (FA), glycerolipids (GL), phospholipids (PL), sphingolipids (SP) and sterols (ST).

As displayed in Figure 2a, four out of five donors showed an increase in total lipid content/mg of tissue in MFAT vs. the original LA, with a total mean increase of about 37%. Only one donor showed the opposite behavior, probably due to a particularly high content of lipids in LA, because the final MFAT content was in line with that of other subjects. Then, we evaluated the contribution of each lipid class and subclass to the total lipid content. Glycerolipids (GLs) and phospholipids (PL) were the two main constituents of both LA and MFAT, accounting for more than 90% of the lipid composition of both matrices (Figure 2b). Although the relative proportions of GL and PL appeared similar between the two materials at first glance, a clear redistribution of GL subclasses was observed in MFAT. In fact, a rise in diacylglycerols (DG) and triacylglycerols (TG) at the expense of oxidized triacylglycerols (OxTG) was observed. Moreover, when investigating the amount of lipids in each lipid class, a statistically significant increase in the GL and PL lipid content of MFAT in respect to the LA counterpart was evident (Figure 2c).

### 3.2. Composition of Lipid Subclasses

**Figure 3:**
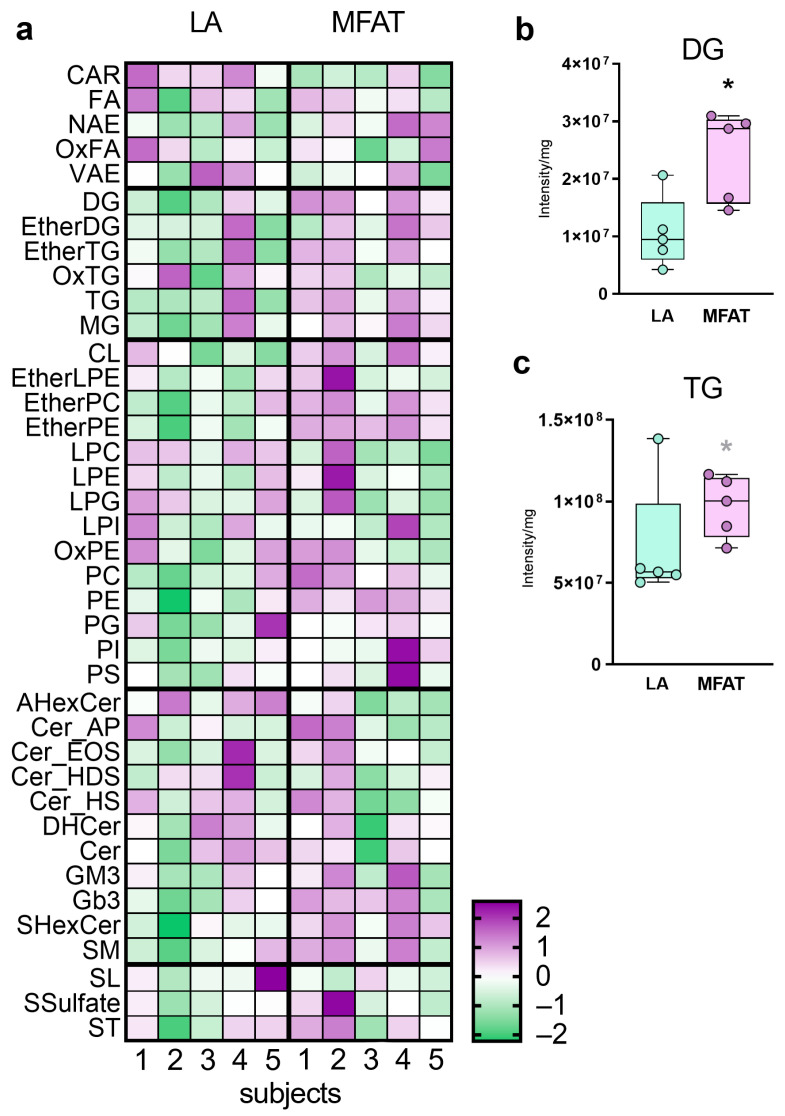
(**a**) Heatmap of the lipid subclass (row) reordered in each super-class (divided by a thick line) content between lipoaspirate (LA), and fat micro-fragmented through the Lipogems commercial device (MFAT). Each column represents a different subject (LA samples on the left and MFAT samples on the right). For visualization, lipid class intensities were Log-transformed and calculated as z-scores. The color-scales differentiate values as average (white), higher (lilac), and lower than average (green). Two representative, differentially expressed and distinctive classes are diacylglycerols (**b**) and triacylglycerols (**c**), whose contents are compared between LA and MFAT. The significance of the difference between TG in LA and MFAT is reached only by excluding the LA-outlier, sample #4. Graphs represent the summed amounts (expressed as mass intensities), after normalization for the mg of fat, of the individual lipids per class (intensity, mean ± SD). Paired *t*-tests were used in each lipid class to establish statistical differences (* *p* < 0.05). Grey * means that the significance of the difference between TG in LA and MFAT is reached only by excluding the LA-outlier sample #4.

Nonetheless, there are more subtle changes in each lipid class, as displayed in the Figure 3a heatmap; once again, increases in GL and PL subclasses in almost all samples, but also in sphingolipids (SL), especially ceramides (, gangliosides (GM3), and sphingomyelins (SM) were observed. Firstly, Figure 3 shows, in detail, the differences between LA and MFAT in the contents of two of the most abundant subclasses of GL (DG 3b and TG 3c). Both DG and TG show an increase in concentration in MFAT that exceeds the initial concentration in LA by more than two-fold.

Since glycerol lipids, especially DG and TG, were augmented in MFAT, we investigated whether the distribution of their pool of fatty acids would be influenced by the respective treatments. The specific extraction of TG from the samples was demonstrated to be very efficient: the phospholipid contamination was confirmed to be uninfluential, accounting for less than 4% (estimated by the quantification of DPPC m/z 734.6 > 184.1, [28]).

**Figure 4:**
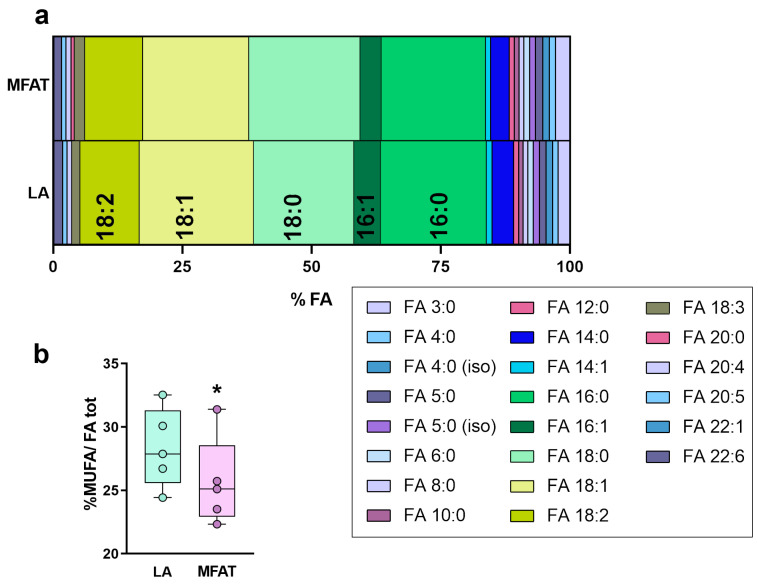
(**a**) Distribution of fatty acids (as percentage of the total) derived from the hydrolysis of triacylglycerols in lipoaspirate (LA), and fat micro-fragmented by a commercial device (MFAT). (**b**) Percentage of monounsaturated fatty acids (MUFA) in total fatty acids derived from the hydrolysis of triglycerides in LA and MFAT. Paired *t*-tests were used in each lipid class to establish statistical differences (* *p* < 0.05).

In general, the distribution of the fatty acids esterified in TG was not significantly modified by the procedure for obtaining MFAT from LA (Figure 4a). Over 50% of their composition consisted of palmitic (20%), stearic (20%), oleic (20%), and linoleic (11%) acids. On that note, a slight decrease in the percentage of the monounsaturated fatty acids in MFAT vs. LA was observed, in particular in the % of long-chain fatty acids (FA 14:1—1.09 vs. 1.00%; FA 16:1—5.10 vs. 4.0%; *; FA 18:1—22.1 vs. 20.5 *). Then, considering the pool of MUFA in TG, the MFAT samples showed a significant decrease in less than 1% of their concentrations (28.3 vs. 27.5%, *, Figure 4b).

## 4. Discussion

Micro-fragmented adipose tissue (MFAT) is easily obtained through abdominal subcutaneous liposuction using different devices and is widely used in regenerative medicine, with particular importance in orthopedic surgery in both animals and humans [29,30,31]. Only a few studies have investigated MFAT biological properties in terms of levels of extracellular matrix content, replicating cells, and stemness-related gene expression [32,33]. No studies exist on the lipidomic pattern of MFAT and its differences to native adipose tissue. Our study investigated adipose tissue obtained from the abdomen, which is a source widely used in regenerative medicine and has a lipid composition largely similar to that of the back and breast area but is significantly different to that of thigh subcutaneous adipose tissue [33]. When comparing LA and MFAT, it can be seen that the preservation of tissue architecture of the MFAT [15] and the trophic and regenerative qualities are very important, which is unlike adipose tissue harvested according to the “standard” technique [34]. According to some studies, the processing of LA into MFAT results in a tissue structure that, after injection, clinically improves the treatment; in particular, this is the case for knee osteoarthritis, as documented by the clinical trials that are currently ongoing [35]. The exact mechanism of action of MFAT and its interaction with the osteoarthritic microenvironment are not fully understood, but it is supposed to be related to soluble factors and EVs, including exosomes [36]. Some authors have reported the importance of MFAT biology with regard to the expression of trophic factors, which improve tissue viability and support the potential benefits of MFAT treatment in inflammatory diseases [37,38]. However, the roles played by lipids should also not be overlooked, because the lipid composition has a role in modulating several cellular processes and intercellular communication, as evidenced during both wound healing and tissue regeneration. Notably, certain bioactive lipids—such as phospholipids—have been shown to attract immune cells and mediate inflammatory responses [39]. As the dysregulated synthesis of phospholipids is a characteristic of the senescent phenotype of MSCs, in aged individuals, wound healing and tissue regeneration could be impaired and delayed due to cell senescence accumulation. Therefore, using MFAT preparation with increased phospholipids could help healing in wound treatments. Another important aspect to consider is the use of MFAT as a scaffold for drug delivery because of its natural character with a well-structured organization capable of releasing anti-tumorigenic concentrations of pre-absorbed chemotherapeutics [22,23]. In particular, as recently reviewed, MFAT could have an interesting role in drug-loading for the treatment of neurological cancer [40]. Our study was designed with the primary aim of providing a detailed lipidomic characterization of MFAT, obtained using one of the most widely adopted systems in clinical practice—Lipogems^®^—which is FDA-approved and certified by the Istituto Superiore di Sanità (Italian Health Authority) and the National Transplant Center, confirming its established safety and regulatory recognition. This initial focused approach represents a necessary step toward establishing a reference point for future comparative studies, which we intend to pursue in future research.

By interpreting the observed lipidomic differences between LA and MFAT, we hypothesize that these variations may result from a combination of (i) the removal of non-adipose components, (ii) improved accessibility to lipid-rich compartments due to tissue micro-fragmentation, and (iii) donor-dependent biological variability.

### Limits and Strengths of the Study

This is a pilot study based on a limited number of subjects. Other studies with larger sample sizes and numbers of fat donors are needed to validate the reported differences between LA and MFAT from a broader perspective.

Another limit of this study is that the lipidomic data were not corrected for the use of a deuterated IS. Additional experiments will be scheduled to include this addition.

The strength of this study is that, for the first time, modifications caused by the MFAT preparation on fat tissue are described.

The results herein presented could serve as a starting point in future studies based on targeted LC-MS/MS with the aim of fully characterizing MFAT for use as a personalized scaffold for drug delivery.

## 5. Conclusions

In conclusion, our study demonstrated, for the first time, that in MFAT, the concentration of different lipid subclasses such as diacylglycerols, triacylglycerols, and sphingolipids was increased. Of course, the biological importance of these findings needs to be better investigated; however, they can contribute to stimulating new studies and aid in better understanding the clinical efficacy of MFAT as well as highlight its potential improvements, not only in regenerative medicine but also as a scaffold for drug delivery.

## Data Availability

The data that support the findings of this study are available from the corresponding authors, C.M. and F.P.

## Abbreviations

The following abbreviations are used in this manuscript:

CAR	Acylcarinitine
Cer-AP	Ceramide alpha-hydroxy fatty acid-phytospingosine
Cer-EOS	Ceramide Esterified omega-hydroxy fatty acid-sphingosine
Cer-HDS	Ceramide hydroxy fatty acid-dihydrosphingosine
Cer-HS	Ceramide hydroxy fatty acid-sphingosine
Cer-NS	Ceramide
CL	Cardiolipin
DG	Diacylglycerol
DHCer	Dihydroceramide
EtherLPC	Ether-linked lysophosphatidylcholine
EtherLPE	Ether-linked lysophosphatidylethanolamine
EtherPC	Ether-linked phosphatidylcholine
EtherPE	Ether-linked phosphatidylethanolamine
EtherTG	Ether-linked triacylglycerol
FA	Fatty acid derivative
GL	Glycerolipid
LA	Lipoaspirate
LPC	Lysophophatidylcholine
LPE	Lysophosphatidylethanolamine
MFAT	Micro-fragmented adipose tissue
MG	Monoacylglycerol
MSCs	Mesenchymal stem cells
NAE	N acyl ethanolamine
NAGly	N acyl glycine
NAGlySer	N acyl glycil serine
NAOrn	N acyl ornithine
OxTG	Oxidized triglyceride
PC	Phosphatidylcholine
PE	Phosphatidylethanolamine
PI	Phosphatidylinositol
PL	Phospholipid
PS	Phosphatidylserine
SHexCer	Sulfatide
SM	Sphingomyelin
SP	Sphingolipid
ST	Sterol lipid
TG	Triacylglycerol
